# Supplementary material for: The interactome of intact mitochondria by cross-linking mass spectrometry provides evidence for coexisting respiratory supercomplexes
Source: Mol Cell Proteomics. 2017 Dec 8;17(2):216–32. doi: 10.1074/mcp.RA117.000470 (PMC5795388; doi:10.1074/mcp.RA117.000470)
Supplement: Supplemental Data [file supp_RA117.000470_133922_0_supp_23978_fzffwf.zip › spectra_annotation/mito_spectra_annotation/247-1-4-1-9-1.pdf]

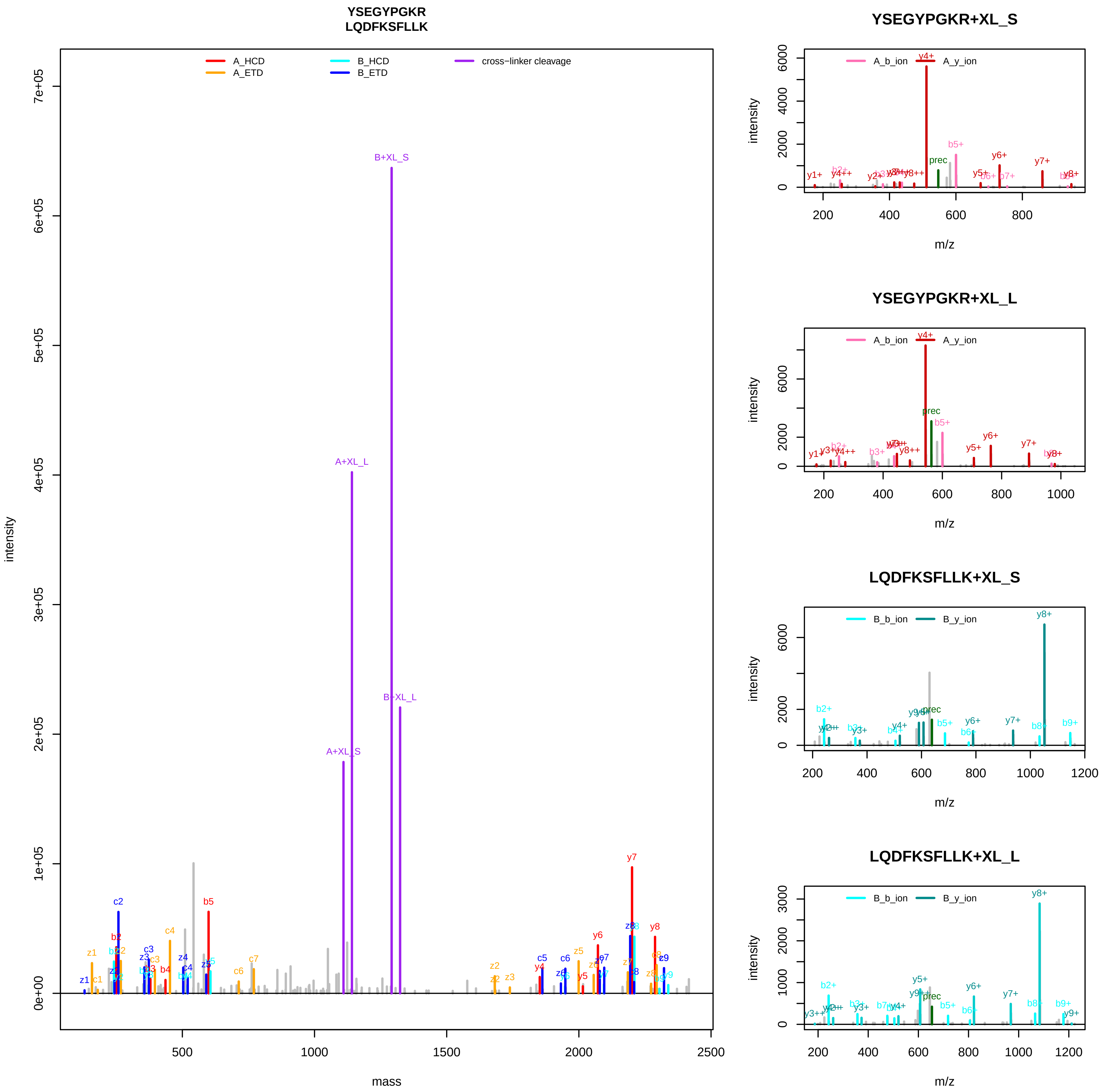
YSEGYPGKR
LQDFKSFLLK
A_HCD
A_ETD
B_HCD
B_ETD
cross−linker cleavage
B+XL_S
A+XL_L
B+XL_L
A+XL_S
intensity
mass
0e+00
1e+05
2e+05
3e+05
4e+05
5e+05
6e+05
7e+05
500
1000
1500
2000
2500
y7
y8
y6
y5
y4
b5
b4
b2
c2
c3
c4
c5
c6
c7
c9
z1
c1
z3
z4
z5
z8
YSEGYPGKR+XL_S
A_b_ion
A_y_ion
y4+
b5+
prec
y6+
y7+
y5+
y1+
y2+
y8+
m/z
YSEGYPGKR+XL_L
LQDFKSFLLK+XL_S
B_b_ion
B_y_ion
y8+
b2+
b5+
y6+
y7+
b9+
LQDFKSFLLK+XL_L
y5+
b8+
y9+
